# The transcription factor STE12 influences growth on several carbon sources and production of dehydroacetic acid (DHAA) in *Trichoderma reesei*

**DOI:** 10.1038/s41598-024-59511-8

**Published:** 2024-04-26

**Authors:** Miriam Schalamun, Wolfgang Hinterdobler, Johann Schinnerl, Lothar Brecker, Monika Schmoll

**Affiliations:** 1 AIT Austrian Institute of Technology GmbH, Center for Health and Bioresources, Konrad Lorenz Strasse 24, 3430 Tulln, Austria; 2 MyPilz GmbH, Wienerbergstrasse 55/13-15, 1120 Vienna, Austria; 3 Department of Botany and Biodiversity Research, University of Vienna, Rennweg 14, 1030 Vienna, Austria; 4 Department of Organic Chemistry, University of Vienna, Währinger Strasse 38, 1090 Vienna, Austria; 5 Division of Terrestrial Ecosystem Research, Department of Microbiology and Ecosystem Science, University of Vienna, Djerassiplatz 1, 1030 Vienna, Austria

**Keywords:** *Trichoderma*, *Hypocrea*, Secondary metabolism, Dehydroacetic acid, Light response, Cellulases, Trichodimerol, Sorbicillin, Transcription factor, Gene expression, Gene regulation, RNA sequencing, Fungal biology, Fungal genetics, Fungal genomics, Fungal physiology, Mass spectrometry, NMR spectroscopy

## Abstract

The filamentous ascomycete *Trichoderma reesei*, known for its prolific cellulolytic enzyme production, recently also gained attention for its secondary metabolite synthesis. Both processes are intricately influenced by environmental factors like carbon source availability and light exposure. Here, we explore the role of the transcription factor STE12 in regulating metabolic pathways in *T. reesei* in terms of gene regulation, carbon source utilization and biosynthesis of secondary metabolites. We show that STE12 is involved in regulating cellulase gene expression and growth on carbon sources associated with iron homeostasis. STE12 impacts gene regulation in a light dependent manner on cellulose with modulation of several CAZyme encoding genes as well as genes involved in secondary metabolism. STE12 selectively influences the biosynthesis of the sorbicillinoid trichodimerol, while not affecting the biosynthesis of bisorbibutenolide, which was recently shown to be regulated by the MAPkinase pathway upstream of STE12 in the signaling cascade. We further report on the biosynthesis of dehydroacetic acid (DHAA) in *T. reesei*, a compound known for its antimicrobial properties, which is subject to regulation by STE12. We conclude, that STE12 exerts functions beyond development and hence contributes to balance the energy distribution between substrate consumption, reproduction and defense.

## Introduction

As for all living beings, reproduction, defense and nutrient acquisition are crucial for survival and competitiveness of fungi in nature. Thereby, balancing resources among these essential tasks in order to optimize colonization and proliferation in their habitat is essential. Diverse signal transduction pathways contribute to this task by integrating sensed environmental cues, rating their relevance under the current conditions and triggering a precisely adjusted output. Fungi of the genus *Trichoderma* are particularly successful in adaptation and competition and are found almost ubiquitously on earth^1^.

The filamentous ascomycete *Trichoderma reesei* represents a model organism for regulation of plant cell wall degradation^2,3^ due to its highly efficient cellulase system^4,5^. The balance between different environmental cues and their relevance as well as regulatory interconnections are subject to research towards signal transduction pathways. Strong connections were observed for light response and regulation of plant cell wall degradation^6^, but also secondary metabolism is influenced by light and carbon sources^7–9^ as is sexual development^10,11^.

STE12 and STE12-like transcription factors are unique to fungi and well-known as targets of the mating/pheromone MAPkinase pathway^12,13^. STE12 and homologous transcription factors are involved in regulation of development^13^ and pathogenicity^14^ in numerous fungi, indicating a well-conserved role.

Moreover, they were suggested to support environmental adaptation^15^. Accordingly, *T. atroviride* STE12 considerably influences growth on diverse carbon sources^16^.

In *S. cerevisiae*, the Kss1 MAPkinase pathway exerts differential expression by binding-imposed repression and phosphorylation dependent activation together with distinct STE12-containing complexes^17^. In yeast, STE12 represents an important node in invasive growth response and mating^18^. Its dual function in these processes served as a model for investigation of signaling specificity to discriminate between pheromone signals and nutrient limitation^12,19^. Interestingly, investigation of the evolution of STE12 and its regulatory functions revealed, that the specific interaction with DNA binding sites evolved in some species and in another lineages only indirect interaction via a binding partner occurs^20^.

Activity of STE12 is predominantly controlled at the posttranslational level via phosphorylation, protein stability and protein–protein interactions in yeast^12^, which is likely also the case in filamentous fungi. In many cases the zinc finger domains are dispensable for DNA binding, while the homeodomain is required^21^.

In plant pathogenic fungi, the ability to penetrate the plant cell wall is crucial for virulence, which requires elevated turgor pressure and accumulation of glycerol. Additionally, nutrient sensing and plant sensing is required for communication and adaptation. In *Fusarium graminearum*, the up-stream MAPkinase targeting STE12 was found to impact activity of extracellular endonuclease, xylanolytic and proteolytic enzymes^22,23^. A negative effect on specific cellulase activity and *cbh1* transcript abundance was found for *T. reesei* TMK1 under controlled light conditions in darkness^24^, although no effect was observed in a previous study for TMK1 under uncontrolled conditions^25^. For *F. graminearum* STE12 a positive regulation of cellulase and protease activities was detected, which is proposed to contribute to pathogenicity^22^. The biosynthesis of the mycotoxin deoxynivalenol (DON) is not affected by deletion of *ste12* in *F. graminearum*^22^.

In *Trichoderma* spp. STE12 was shown to act downstream of the TMK1 MAPkinase cascade and influences mycoparasitism, hyphal avoidance, vegetative hyphal fusion, expression of cell wall degrading enzymes relevant for mycoparasitism and carbon source dependent growth of *T. atroviride*^16^. Consequently, we were interested in functions of STE12 in gene regulation upon growth on cellulose, the most important carbon source in the natural habitat of *Trichoderma*, as well as its role in carbon utilization and secondary metabolism.

## Results

### The domain structure of STE12 in filamentous fungi is conserved in T. reesei

**Figure 1:**
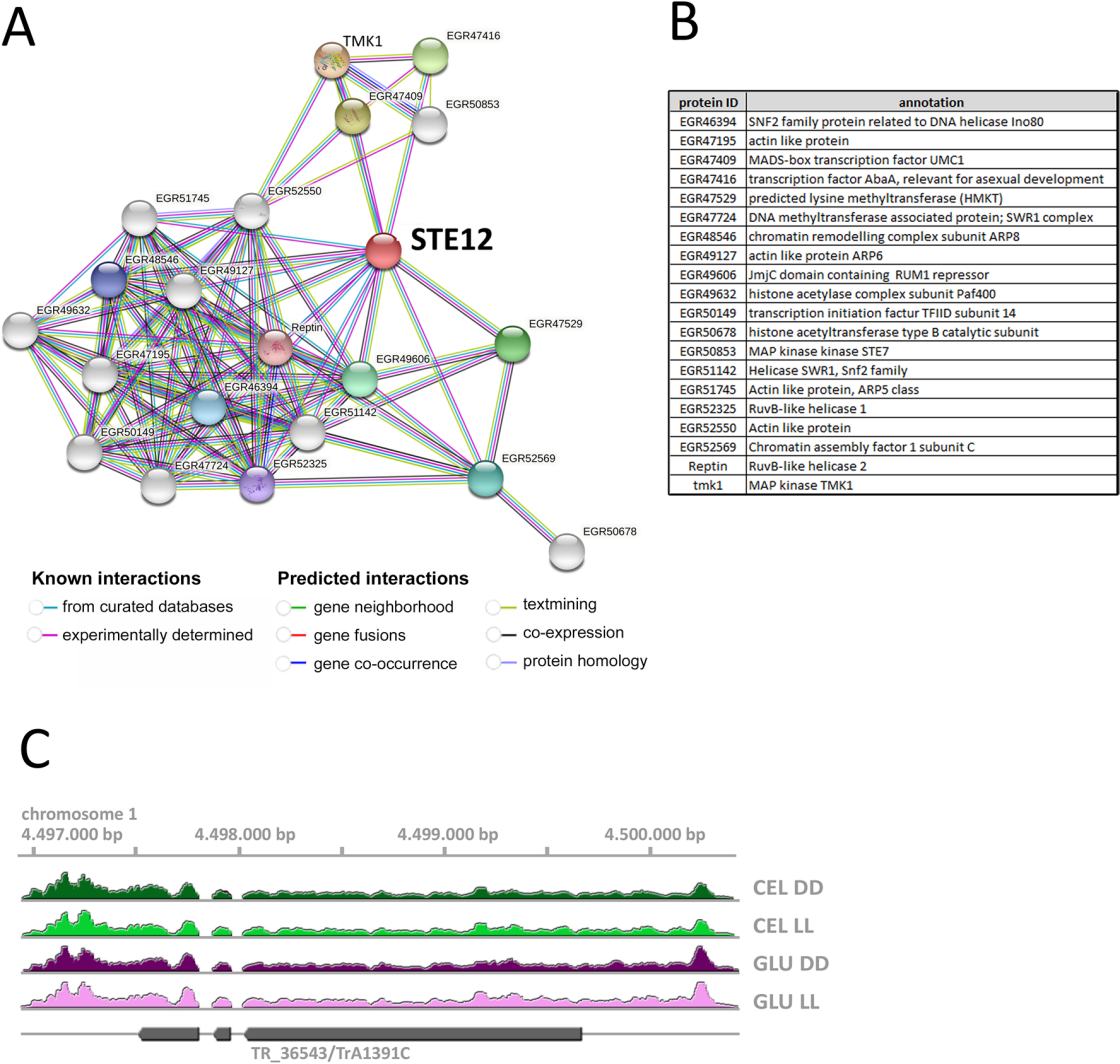
Characteristics of *ste12*/TR_36543/TrA1391C and its encoded protein. (**A**) STRING network of known and predicted interaction partners. The network for interactors of STE12 was drawn using the online STRING search function (https://string-db.org/) in version 12^91^ (**B**) Annotations of predicted interaction partners of STE12. (**C**) Evaluation of the protein model of STE12 by analysis of aligned reads from available transcriptome data for growth on cellulose (CEL) or glucose (GLU) in constant light (LL) or constant darkness (DD).

In order to integrate STE12 in the context of current network knowledge, we searched for known and predicted interactors using the STRING database (Fig. 1A). The protein interaction network of STE12 in *T. reesei* revealed a connection to the mating related MAPkinase pathway and TMK1, as well as numerous genes involved in chromatin modification (Fig. 1B). Since *ste12* homologues were previously reported to be subject to alternative splicing^26,27^, we screened available transcriptome data for coverage of the *ste12* gene model, which contains two introns. For evaluation of the gene model used in our analysis for *ste12*, we checked data from growth on cellulose or glucose in constant light or darkness (Fig. 1C). We found that the predicted introns are clearly present and that *ste12* has a relatively long 5′ UTR of roughly 700 bp, which comprises an upstream in-frame stop codon at position − 24. In this UTR region, neither an additional intron nor an upstream open reading frame (uORF;^28^) was detected, which might interfere with efficient *ste12*-translation. No indications for alternative splicing were detected.

The fungal STE homeodomain is highly divergent, however, in this domain also a conserved stretch of KQKVFFWFSVA resides^29^. Indeed, a related sequence is also present in *T. reesei*, albeit with three amino acid alterations: KQKVFYWYSVP. Accordingly, *T. reesei* STE12 comprises a STE like transcription factor domain (pfam02200; p-value 1.17e-78).

### STE12 positively influences cellulase gene transcription

**Figure 2:**
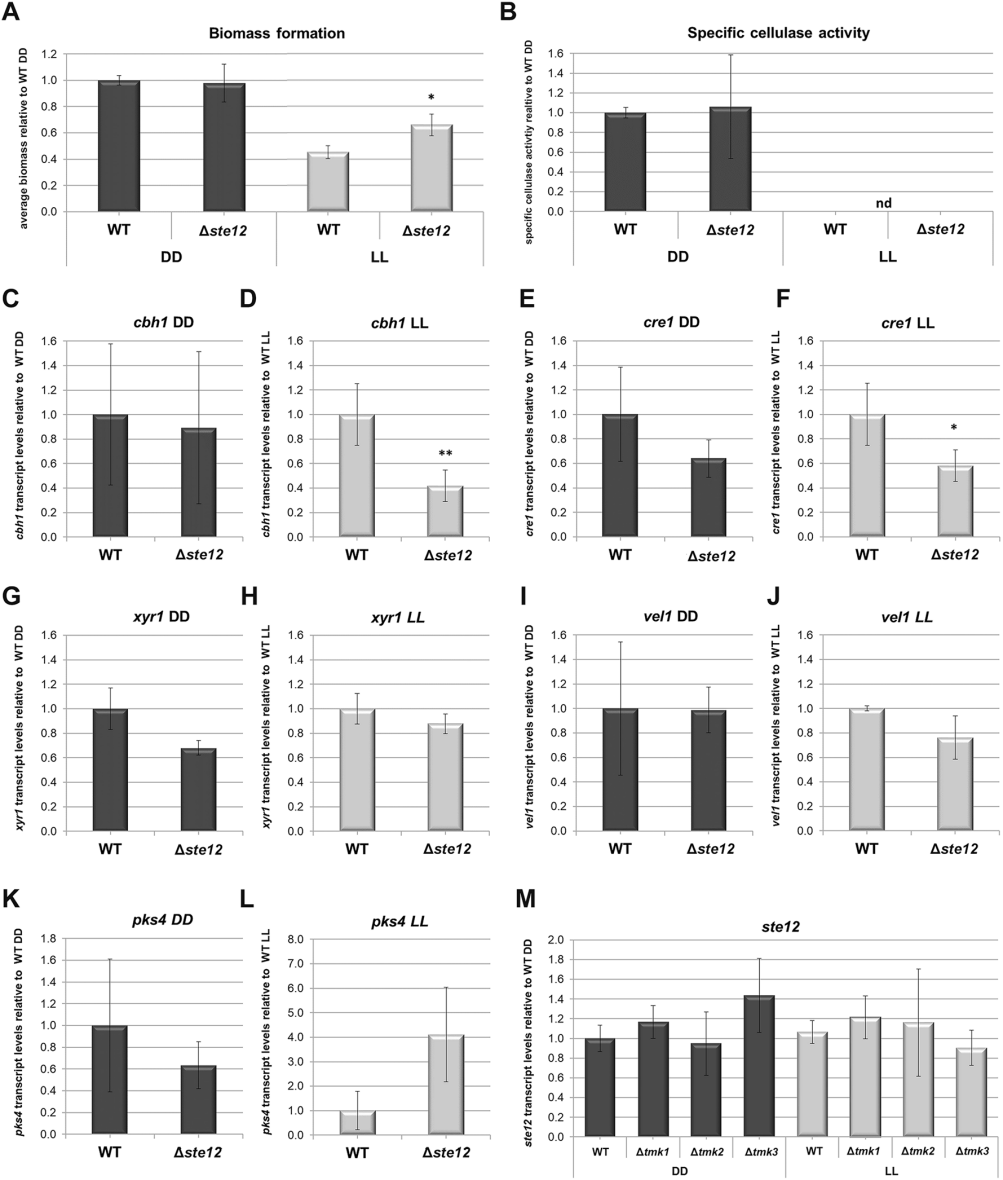
Relevance of STE12 for biomass formation, cellulase activity and gene regulation upon growth on 1% cellulose. (**A**) Biomass formation relative to wild-type (WT). (**B**) Specific cellulase activity. (**C**, **D**) Transcript levels of *cbh1* (**C**) in constant darkness and (**D**) constant light. (**E**, **F**) Transcript levels of *cre1* (**E**) in constant darkness and (**F**) constant light. (**G**, **H**) Transcript levels of *xyr1* (**G**) in constant darkness and (**H**) constant light. (**I**, **J**) Transcript levels of *vel1* (**I**) in constant darkness and (**J**) constant light. (**K**, **L**) Transcript levels of *pks4* (K) in constant darkness and (**L**) constant light. (**M**) Transcript levels of *ste12* in MAPKinase deletion mutants in constant darkness (DD) and light (LL).

Interestingly, *ste12* shows mutations in the *T. reesei* cellulase-mutant strains NG14 and RutC30^30,31^, suggesting a potential contribution to the efficient production of cellulose degrading enzymes in these strains. We asked whether STE12 has a function in regulation of cellulase formation upon growth on cellulose. Therefore, we first tested whether biomass formation on this carbon source would be altered. In darkness, Δ*ste12* showed similar growth as the wild-type, while in light biomass formation was significantly increased by 20% (Fig. 2A). However, while specific cellulase activity in darkness was unaltered and hence consistent with growth data, activity in light remained below detection levels (Fig. 2B). Analysis of *cbh1* transcript abundance showed a positive effect of STE12 in light, causing a decrease of *cbh1* transcript by 60% upon *ste12* deletion but no effect in darkness (Fig. 2C, D). A similar effect was detected for the carbon catabolite repressor gene *cre1*, with a 40% decrease in transcript abundance for Δ*ste12* in light (Fig. 2E, F). For the cellulase transcription factor gene *xyr1*, no significant regulation by STE12 was found, although in darkness a negative trend of transcript levels was apparent (p-value 0.071) (Fig. 2G, H). The important regulatory gene *vel1*, which is required for cellulase induction^32^ and impacts secondary metabolism also in *T. reesei*^33,34^ is not significantly regulated by STE12 (Fig. 2I, J). *Pks4*, the gene encoding the polyketide synthase responsible for the green pigment in spores of *T. reesei*^35^ shows a trend towards increased abundance in the mutant in constant light, albeit the respective p-value (0.092) is below our threshold for significance set at 0.05 (Fig. 2K, L). The same upregulation is observed for gene expression analysis by RNA-sequencing, which resulted in a significant five-fold upregulation of *pks4* in constant light for Δ*ste12,* confirming the validity of the sequencing results.

Of the five MAPkinase cascades of *S. cerevisiae*, two, Fus3 and Kss1 target Ste12^12^ to transmit the pheromone signal. Since STE12 is subject to regulation by MAPkinases also in other fungi^16,36,37^, although predominantly in terms of phosphorylation and stability, we asked whether in *T. reesei* also effects on the transcriptional level are present. Our analysis showed that *T. reesei ste12* is not subject to transcriptional regulation by MAPkinases upon growth on cellulose (Fig. 2M).

### Growth on different carbon sources is altered in Δste12

A more general role of STE12 in regulation of growth and hence metabolism on diverse carbon sources was investigated using the BIOLOG phenotype microarrays^38^. We monitored growth patterns from 72 to 144 h after inoculation in constant darkness (supplementary file 1). If two consecutive time points showed statistically significant differences (p-value < 0.05, t-test) in biomass formation as analyzed by turbidimetry at 750 nm, we considered STE12 to be relevant for regulation of growth on this carbon source.

**Figure 3:**
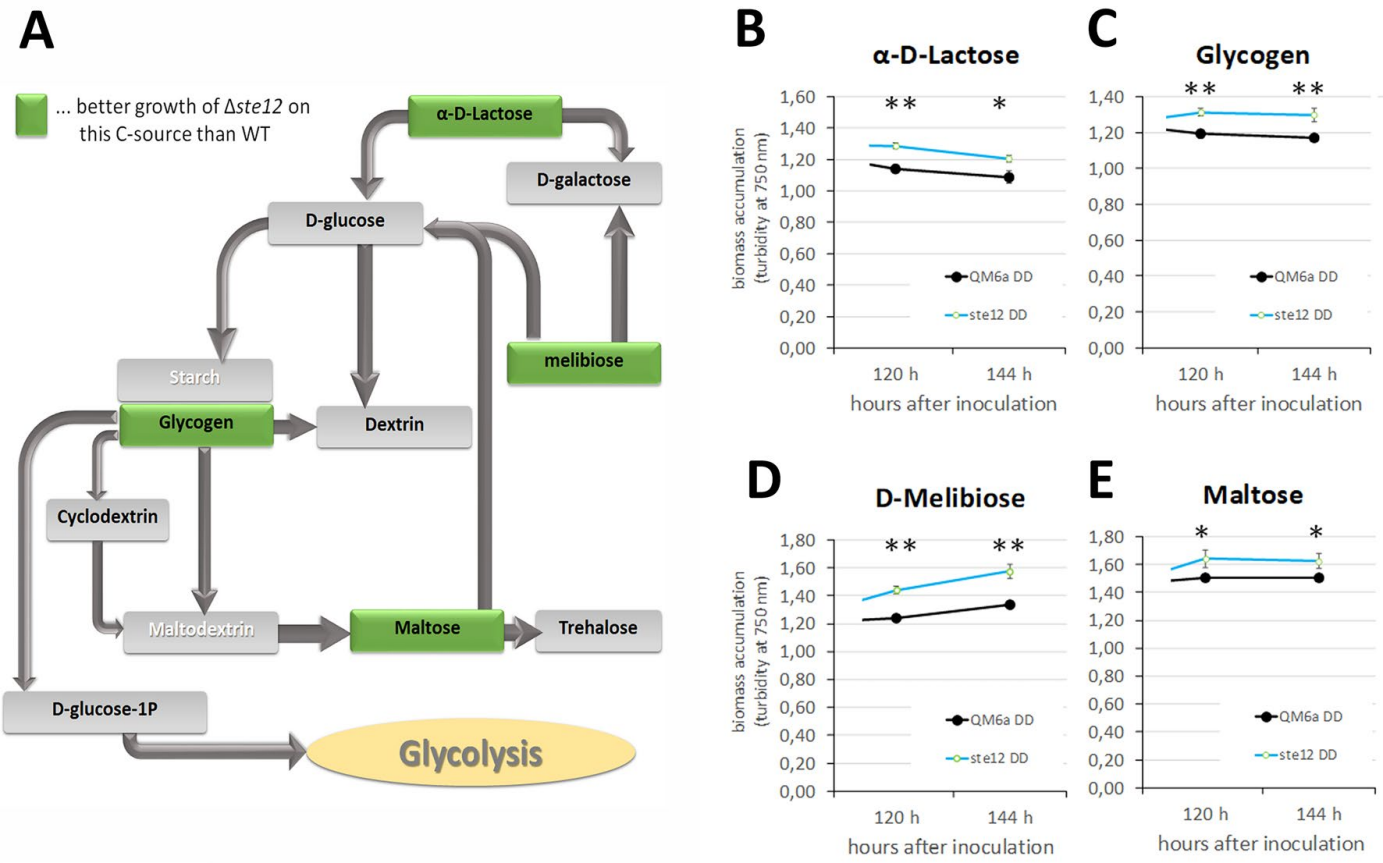
Analysis of carbon source utilization using the BIOLOG phenotype microassay. (**A**) Schematic representation of carbon sources on which Δ*ste12* grows better than the wild-type along with conversion pathways as deduced from KEGG pathways for *T. reesei*. (**B**, **C**) Growth data of the *ste12*-deletion strain as represented by turbidimetric analysis of biomass accumulation at 750 nm and compared to the wild-type strain QM6a. The analysis was done in biological triplicates with growth in darkness (DD). Statistical significance was determined by the T-test; * = p-value < 0.05, ** = p-value < 0.01.

Interestingly, the differences we found for Δ*ste12* were all positive in terms of elevated growth of the mutant strain compared to the wild-type strain (Fig. 3A–E). In many cases, these differences occurred at 120 and 144 h after inoculation, when the mutant strain obviously kept growing, whilst the parental strain did not. Better growth on glycerol and glycogen suggests utilization of these carbon sources instead of storage.

Moreover, sugars including lactose, lactulose, melibiose, maltose and melezitose enable longer growth of the mutant strain, as do γ-hydroxy butyric acid, *p*-hydroxyphenylacetic acid and α-keto-glutaric acid (Fig. 3A–E).

### STE12 impacts gene regulation

**Figure 4:**
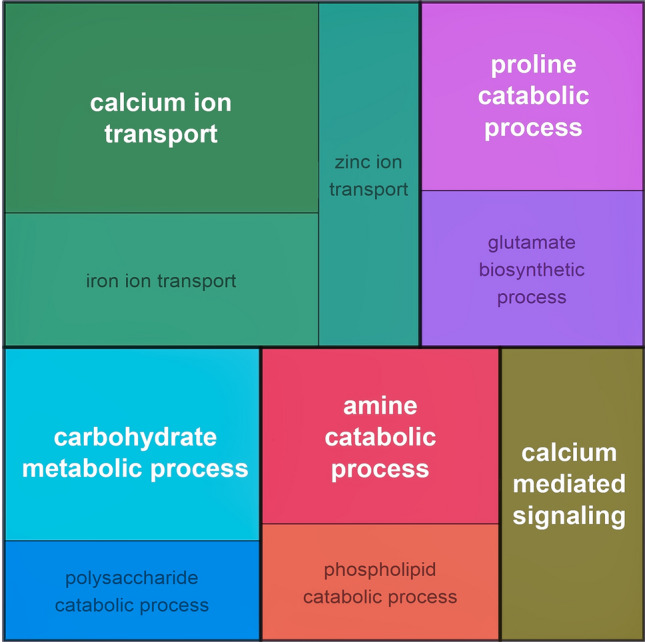
Gene ontology analysis of genes regulated by STE12. GO enrichment of up- and down-regulated genes for Δ*ste12* in constant darkness and light, visualized with rrvgo in R.

We investigated the regulatory impact of STE12 on gene regulation upon growth on cellulose as carbon source in constant light and constant darkness. In total, we found 203 genes to be more than twofold significantly (p-adj < 0.05) regulated directly or indirectly by STE12 (Supplementary file 2). Functional category analysis (supplementary file 2) of these genes revealed a significant enrichment (p-value < 0.05) of genes involved in transport facilities, particularly calcium-, iron- and zinc-ion transport, carbohydrate metabolic process as well as secondary metabolism (amine- and proline catabolic process). Gene ontology (GO) analysis supported these results (Fig. 4).

Of the STE12 targets, 84 genes were up-regulated in light, including nine CAZyme encoding genes, for example a candidate chitinase (TrA0008W/TR_59791, 28.8-fold) and a subgroup beta-chitinase (TrE0823C/TR_43873, 8.8-fold), a candidate glycoside hydrolase (TrC0858W/TR_55886) and a beta-xylosidase, *bxl1* (TrC1552C/TR_121127). Other upregulated genes include the conidiation specific *con-10,* TrD0147C/TR_5084 (11.7-fold), the protein kinase gene *gin4* (TrD1202W/TR_64125*)*, which positively influences trichodimerol biosynthesis^39^ a candidate cutinase transcription factor (TrA0431C/TR_106259, 26.3-fold). TR_106259 is also strongly up-regulated in a deletion mutant of the secondary metabolite regulator of the SOR-cluster, YPR2^8,40,41^, corroborating an indirect effect of STE12 on secondary metabolism. The same applies for a second strongly up-regulated transcription factor gene, TrF0487C/TR_112643 (12.7-fold in darkness), which is also strongly up-regulated in Δ*ypr2*^41^. Further up-regulated genes include the gene encoding the glucose transporter HXT1 (TrE0206W/TR_22912), the predicted sugar transporter gene TrD0036W/TR_50894, which was shown not to be required for growth on lactose^42^ along with several other transporter genes as well as two genes encoding proteins predicted to be involved in plant surface sensing^43^, the effector protein encoding TrA1330W/TR_72907 and the PTH11 type G-protein coupled receptor gene TrG0742C/TR_45573. Among the up-regulated genes in light, four genes belong to the cytochrome p450 superfamily, where TrF0040C/TR_65036 and TrA1084W/TR_75713 are potential homologues of *Aspergillus nidulans* alkane hydroxylases, catalyzing the oxidation of alkanes. The other two cytochrome p450 encoding genes are TrE0324C/TR_66453 homolog of *N. crassa ci-1*, an *ent*-kaurene oxidase, involved in the biosynthesis of gibberellins^44^ and TrA0963W/TR_67377. Additionally, two polyketide synthase genes, *pks4* (TrD1440W/TR_82208), responsible for the green pigmentation of *T. reesei* conidia^35^ and *pks2* (TrD0448W/TR_65891) are up-regulated (5- and 2.8-fold) in light.

The 23 genes up-regulated in darkness comprise the conidiation associated glucose repressible gene *grg-1,* TrE0533C/TR_73516, a family 5 carbohydrate esterase, the xylanase gene *xyn3* (TrF0312W/TR_54219 and TrC0667W/TR_120229), the non-ribosomal peptide synthase (NRPS) encoding *tex2* (TrB1256C/TR_123786) responsible for paracelsin biosynthesis. Furthermore, two mitochondrial transporters TrC0706C/TR_103853 and TrF1000W/TR_121743 and a small cysteine-rich protein encoding gene TrC1533/TR_121135 (90.3-fold).

The 86 genes of the gene set down-regulated in light comprises four CAZyme encoding genes including *cbh1*/*cel7a* (4.1-fold), *egl3*/*cel12a* (23-fold), which is limiting for high efficiency plant cell wall degradation^45^, the beta-glucosidase *bgl1*/*cel3a* (45.2-fold) and a GH 99 gene, TrC1527C, TR_121136 (21.9-fold). Additionally, among the down-regulated genes in darkness are the GprK-like RGS domain containing heterotrimeric G-protein coupled receptor gene TrG0214W/TR_81383 and three transcription factor genes (TrA0076W/TR_3605, TrG1015C/TR_120363 and TrD0324W/TR_80139). The 10 down-regulated genes in darkness include a predicted oligonucleotide transporter gene related to sexual differentiation process protein ISP4 (TrA1796W/TR_124002), and a predicted MFS permease (TrB1842C/TR_68990).

Of all STE12 targets, five genes contain mutations in the high cellulase producer RutC30 (TrB1256C/TR_123786, TrG0579W/TR_56726, TrF0040C/TR_65036, TrF0049W/TR_65039 and TrC0660W/TR_120231).

Plant cell wall degradation specific phosphorylation was detected previously^46^ for six STE12-regulated genes including an amino acid transporter (TrB0212C/TR_123718), *grg-1* and a putative methyltransferase gene (TrD1044C/TR_108914).

### Regulation by STE12 in both light and darkness

Eight genes show light independent regulation by STE12. Up-regulation in both, light and darkness, was observed for a potential amino acid transporter gene (TrB0212C/TR_123718), the polyketide synthase gene *pks2* (TrD0448W/TR_65891), a potential carnitine *O*-acyltransferase encoding gene (TrC0399W/TR_122240) and TrE0645C/TR_54352. The putative exonuclease protein TrA1281W/TR_57424, a siderophore transporter TrG0054C/TR_82017 and TrA1279C/TR_57823 (PRE containing) were down-regulated in light and darkness. One gene, TrD0165W/TR_50793, encoding a putative homologue of QIP, a putative exonuclease protein involved in quelling with contrasting regulation in light and darkness by STE12 was found.

### STE12 influences genes involved in iron homeostasis

Interestingly, several genes involved in iron homeostasis are targeted by STE12: The genes encoding the multicopper peroxidase Fet3b (TrD0040C/TR_5119) and the high affinity iron permease Ftr1b (TrD0041W/TR_80639), both belonging to the reductive iron uptake system^47^, are up-regulated in light in Δ*ste12*. Moreover, a gene encoding a predicted, Fet5 related ferroxidase (TrD1438C/TR_124079) as well as a predicted siderophore transporter (TrD0541W/TR_67026) are upregulated in light. In contrast another siderophore transporter gene (TrG0054C/TR_82017) is downregulated in darkness. Additionally, a predicted iron transporter (TrD0323C/TR_38812) is 11-fold down-regulated in light. These findings suggest a contribution of STE12 to light modulated regulation of iron homeostasis.

### Presence of the pheromone response element (PRE) in STE12 target promotors

The target sequence motif of STE12 was determined in *S. cerevisiae* and is called pheromone response element (PRE): 5′ (A)TGAAACA 3′^29,48^. Multimerization of *S. cerevisiae* Ste12 appears to enhance binding to pheromone response elements (PREs) and several adjacent PREs occur in strongly pheromone induced genes^49,50^, although a clear correlation was not found and pheromone responsive genes without PREs also exist^51,52^.

This sequence is also essential for Ste12 binding in *C. neoformans*^53^ and in *Colletotrichum lindemuthianum*^27^. Screening the genes regulated by STE12 in *T. reesei* on cellulose, we found PREs in the promotors of five target genes (TrE0645C/TR_54352, TrA1206C/TR_104816, TrF0872C/TR_107349, TrA0569C/TR_108586 and TrA0485W /TR_121285). The reverse sequence 5’ TGTTTCA 3’ was present in 14 of the *T. reesei* STE12 target genes (supplementary file 2) CAZyme encoding genes, *grg-1* and a putative amino acid transporter. However, in none of these promotors we found more than one motif or a combination of forward and reverse motifs.

### STE12 regulates production of dehydroacetic acid and trichodimerol

Functional category analysis of genes regulated by STE12 upon growth on cellulose revealed a significant enrichment of genes associated with secondary metabolism among its targets. Moreover, regulation of development is among the primary functions of STE12 in fungi^13,37,54^, which is accompanied with clear alterations in secreted metabolites in *T. reesei*^33^. Consequently, we asked whether STE12 is required for proper chemical communication under conditions favoring sexual development.

**Figure 5:**
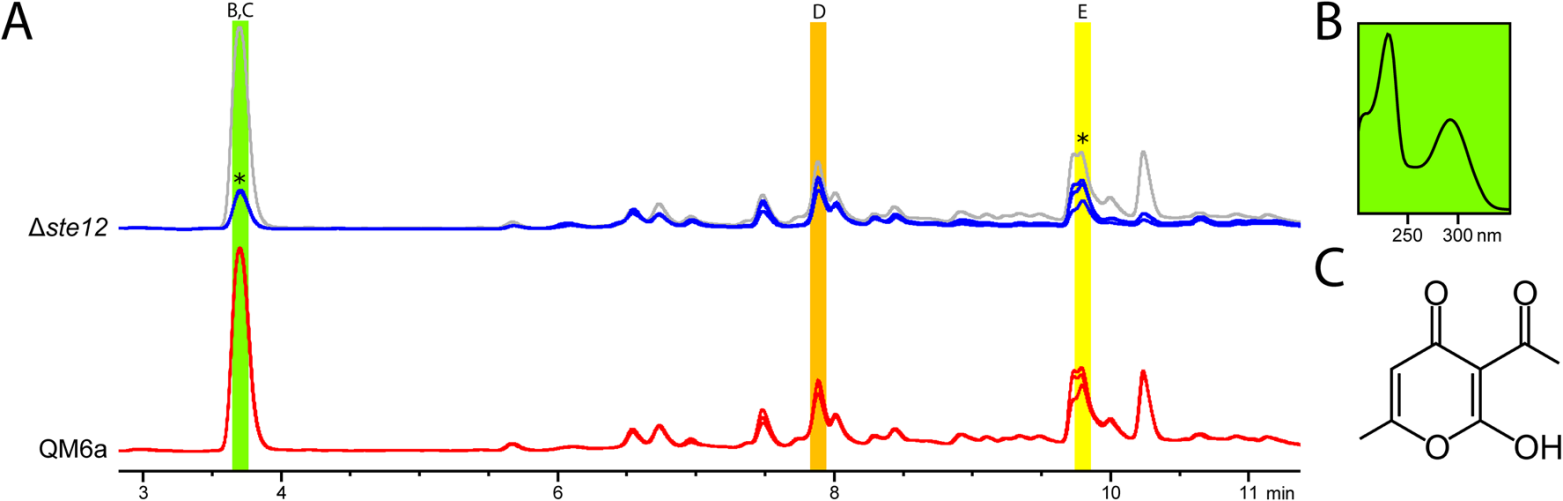
HPLC analysis of secondary metabolite production and identification of dehydroacetic acid. (**A**) HPLC–DAD chromatograms of QM6a and Δ*ste12* at 230 nm. QM6a profile is shown in grey for better comparison. Three biological replicates are shown. Strongly regulated peaks are indicated by asterisks. Dehydroacetic acid (B,C) is highlighted in green, (21S)-bisorbibutenolide (**D**) in orange and trichodimerol (**E**) in yellow^24^. (**B**) UV-spectrum and (**C**) chemical structure of dehydroacetic acid.

Bisorbibutenolide, which was recently shown to be produced by *T. reesei* and dependent on the presence of the MAPkinase TMK3^24^, is not regulated by STE12 (Fig. 5A, highlighted in orange (D)). However, STE12 is involved in regulation of dehydroacetic acid (highlighted in green (B, C)) and also trichodimerol (highlighted in yellow (E)) in Fig. 5A.

Preparative column chromatography fractions obtained from *T. reesei* crude extracts were subjected to NMR and MS analysis and resulted in the identification of dehydroacetic acid (Fig. 5B, C). It was identified in a mixture together with the steroid ergosterol (sample A), in a further purified sample (B) and finally by comparison to a commercially available standard.

The NMR spectroscopic analysis of sample A revealed a content of approx. 90% (mol/mol) ergosterol (Fig. S1 in supplementary file 3). These NMR spectroscopic data of ergosterol are in agreement with those of a commercial reference sample as well as with previously published data of ergosterol^55^. In addition, approximately 7% (mol/mol) of the target compound could be identified from the mixture in sample A. Further purification of this smaller amount in sample A by prep TLC using silica gel 60 glass plates (Merck) yielded 0.6 mg of the target compound (sample B). It was identified as dehydroacetic acid (3-acetyl-6-methyl-3,4-dihydro-2H-pyran-2,4-dione, DHAA).

HR-ESI-TOF–MS in negative ionization of sample A (Fig. S2 in supplementary file 3) shows a deprotonated molecular ion [M-H]^-^ of *m/z* 167.0343, which correlates quite well with the calculated [M-H]^-^ of *m/z* 167.0350 of the molecular formula C_8_H_8_O_4_. The HR-ESI-TOF–MS of sample B (Fig. S3, S4 in supplementary file 3) shows a deprotonated molecular ion [M-H]^−^ of *m/z* 167.0349 in the negative ionization mode as well as a [M + Na]^+^ of *m/z* 191.0309 and a [M + H]^+^ of *m/z* 169.0489 in positive ionization mode. The isotopic patterns in these spectra of sample B show a weak entry of deuterium into the molecule, because it was previously dissolved in CD_3_OD. However, all recorded monoisotopic masses fit well with calculated [M-H]^-^ of *m/z* 167.0350, [M + Na]^+^ of *m/z* 191.0315 and a [M + H]^+^ of *m/z* 169.0495 of the molecular formula C_8_H_8_O_4_. Further co-chromatographic comparison using commercially available dehydroacetic acid (Thermo Scientific, Waltham, MA; CAS Nr. 520-45-6) as standard confirmed the identity of this compound in sample B (Fig. 5).

1D and 2D NMR measurements of the 7% (mol/mol) dehydroacetic acid in sample A further confirmed the structure of the target compound (Fig. S5 in supplementary file 3). The spectra led to a total number of two methyl-, zero methylen-, one methine groups and five quaternary carbon atoms, resulting in one additional non carbon bound proton. The ^1^H NMR signal of the methyl group at pos. 8 (δ_H_ 2.58 ppm/δ_C_ 30.7 ppm) shows in HMBC a ^2^*J*_H-C_ coupling to the keto function at C-7 (δ_C_ 206.7 ppm) and a ^3^*J*_H-C_ coupling to the quaternary C-3 (δ_C_ 100.9 ppm). Furthermore, the ^1^H NMR signal of the methyl group in pos. 9 (δ_H_ 2.28 ppm/δ_C_ 21.2 ppm) shows a ^2^*J*_H-C_ to C-6 (δ_C_ 171.7 ppm) and a ^3^*J*_H-C_ on the of the methylene group at C-5 (δ_H_ 6.14 ppm/δ_C_ 102.2 ppm). The corresponding H-5 shows a further ^2^*J*_H-C_ to C-4 (δ_C_ 180.4 ppm), while the ^13^C NMR signal from C-2 cannot be determined in HMBC and is assumed to be as weak signal at 162.4 ppm. All these chemical shifts and couplings are in good agreement with those reported earlier^56,57^. Numbering of protons and carbons as well all chemical shifts and couplings are shown in Fig. S6 in supplementary file 3.

## Discussion

**Figure 6:**
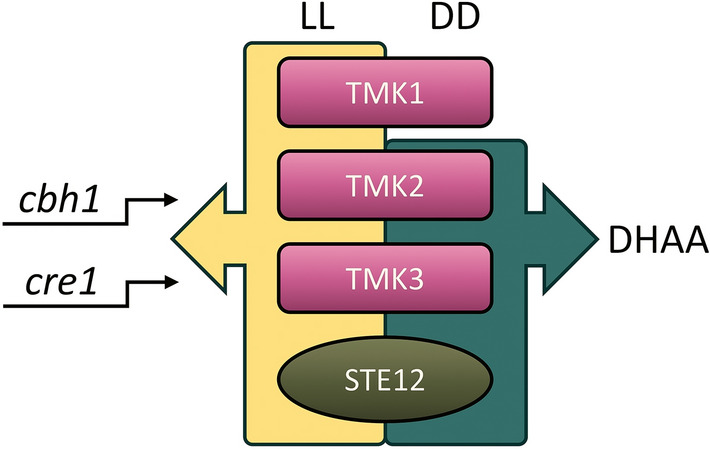
Schematic representation of the light dependent and overlapping targets of the MAPKinase pathway and STE12. The MAPKinases TMK1, TMK2, TMK3^24^ and STE12 influence cellulase production through the consistent up-regulation of the cellobiohydrolase encoding gene *cbh1* and the carbon catabolite repressor gene *cre1* in constant light (LL). In darkness (DD), TMK2, TMK3 and STE12 are required for the biosynthesis of dehydroacetic acid (DHAA).

We explored the role of STE12 in regulation of metabolic pathways, which are crucial to application of the industrial workhorse *T. reesei* (Fig. 6). Ste12 is a transcription factor that was first described in the yeast *S. cerevisiae* where it acts downstream of the mating and invasive growth response pathways which are controlled by the Fus3 and Kss1 MAPkinases respectively^13^. In other *Trichoderma* species like *T. atroviride*, Ste12 is also linked to the Fus3/Kss1 homolog Tmk1, and several Tmk1-mediated processes, including mycoparasitism, hyphal growth, and carbon source utilization, are regulated through Ste12^16^. Hence, we were also interested in overlapping functions of MAPkinases and STE12 in *T. reesei*. Interestingly, we did not detect a regulation of transcript abundance of *ste12* by any of the three MAPkinases in *T. reesei* upon growth on cellulose. Consequently, the MAPkinase cascades either do not regulate STE12 on cellulose or this regulation occurs at a posttranscriptional or posttranslational level.

Given the previous findings indicating that various functions regulated by MAPkinases in *T. reesei* are light-dependent^24^, we regarded light as a critical environmental factor when exploring the role of STE12. Indeed, we found varying gene expression regulation for constant light and constant darkness by STE12 on cellulose, the carbon source closest to its natural habitat. In light, deletion of *ste12* leads to an up-regulation of CAZyme encoding genes, specifically chitinases and glycosidases, which resembles the observations in *T. atroviride* where chitinase encoding genes were upregulated upon growth on chitin^16^. Whereas other CAZyme genes such as the prominent cellobiohydrolase *cbh1* gene and the beta-glucosidase gene *bgl1* are downregulated.

When comparing this regulation in Δ*ste12* to the MAPkinases in *T. reesei*, the pattern of *cbh1* down-regulation in the presence of light aligns with the regulation pattern observed for all three MAPkinases, TMK1, TMK2, and TMK3. This observation suggests a possible involvement of STE12 in the cellulase signal transmission by all three MAPkinases in light, likely at a posttranslational level by phosphorylation. In darkness, however, there is no significant *cbh1* regulation in Δ*ste12* whereas the MAPkinases in this case show contrasting significant regulations, showcasing the complex interplay between signal transmission cascades and environmental cues in cellulase regulation. Similarly, there is an increase in biomass formation upon growth of Δ*ste12* on cellulose in light however in darkness there is no change of growth.

In *Trichoderma*, the green pigmentation of spores is attributed to the activity of polyketide synthase PKS4^35^. In the *T. reesei* Δ*tmk3* mutant, the expression of the *pks4* gene is completely abolished, resulting in spores lacking their characteristic green color^24^. Conversely, when the MAPkinase *tmk2* is deleted, there is a significant increase in *pks4* gene expression in the presence of light^24^, mirroring a similar response observed upon the deletion of *ste12.* Therefore, a contribution of STE12 to transmission of the signal regulating *pks4* by the cell integrity pathway (TMK2) in light would not be without precedent.

Our analysis of carbon source utilization, i.e. growth on diverse carbon sources in darkness, did not reveal dramatic alterations in growth of Δ*ste12*, indicating that STE12 is not essential for the considerable adaptation competence of the metabolism of *T. reesei*. Nevertheless, in several cases, lack of *ste12* appeared to result in better fitness in terms of achieving higher biomass at later time points of growth. We conclude that STE12 is involved in modulation of growth for adaptation to different nutrient conditions in *T. reesei* and that its function is rather a negative one.

Our transcriptome analysis hinted at a contribution of STE12 to regulation of secondary metabolism with respect to siderophore biosynthesis and transport as well as iron transport. Additionally, strong up-regulation of two transcription factors also up-regulated in the absence of an important sorbicillinoid regulating transcription factor, YPR2^41^ indicate a function in secondary metabolism. Previously, STE12 was found to play a role in *Athrobotrys oligospora*, a nematode trapping fungus, in secondary metabolism under trap formation conditions^58^ However, in *Fusarium graminearum*, abundance of the important secondary metabolite DON (deoxynivalenol) was not influenced by STE12^22^, while other compounds were not analyzed in the respective study.

Iron is among the most important nutrients for survival of microbes and hence it is subject to competitive actions^59^. The involvement of STE12 in iron homeostasis and siderophore associated gene regulation was not reported before and is likely specific to growth on cellulose. However, we also want to note here that this effect occurred in light, where the mutant strain grows somewhat better (Fig. 2A) and may hence reach iron-limiting conditions, which facilitate siderophore production^60^, earlier than the wild-type.

We could previously show that the chemical communication with mating partners is not limited to secretion and sensing of peptide pheromones, but involves further secreted metabolites^33^, including the sorbicillin derivative trichodimerol^39^. Regulation of this chemical language in *T. reesei* involves different sensing and signaling factors like protein kinase A^39^, the secondary metabolite regulator VEL1^33^, the photoreceptor ENV1^61^ and the transcription factor SUB1^62^. Since the most thoroughly investigated function of STE12 involves the regulation of development, we figured that under these conditions, also modulations of secondary metabolites, likely including those of sorbicillins should occur.

Sorbicillinoids are by now among the best studied secondary metabolites of *T. reesei*^8,40^. The SOR-cluster, which is responsible for sorbicillinoid production, was acquired by *T. reesei* by lateral gene transfer^63,64^ and is regulated by light^8^. These compounds have anti-inflammatory, cytotoxic and antimicrobial effects^65^. HPLC analyses confirmed the connection of STE12 to sorbicillinoid production with an influence on trichodimerol production under conditions facilitating sexual development (Fig. 5). Interestingly, abundance of bisorbibutenolid, which was recently shown to be produced in *T. reesei*^24^, was not altered, indicating that STE12 acts selectively on production of sorbicillinoids.

The identified compound dehydroacetic acid (DHAA) was recognized already in the nineteenth century as a possible intermediate of the polyketide pathway^66^. It thus belongs to this large group of natural products and is a possible intermediate and building block for larger polyketides^67^. Furthermore, the antifungal effect of DHAA was also recognized in 1947^68^ and led to an industrial production and wide use of this compound. As a result, DHAA can nowadays be found as a contaminant in various places in nature^69^. However, so far only a few reports have been described in which DHAA is isolated and described from natural sources, e.g.^70,71^, including an isolation from *Trichoderma viride*^72^. This lack of reports on isolation may be due to the fact, that DHAA is further converted in the polyketide pathway and hence not further accumulated in many organisms. The regulation of DHAA production by STE12 under conditions facilitating sexual development may hint at a function in adjusting defense during the energy consuming mating process in *T. reesei*. Alternatively, STE12 might regulate expression of enzymes required to convert DHAA to the target-polyketides, which results in accumulation of DHAA depending on the presence of STE12 in the genome.

In summary, we found that STE12 is involved in regulation of transcript abundance upon growth on cellulose and that its function is distinct in light and darkness. Due to the strongly negative impact on two further transcription factors, it can be assumed that STE12 not only acts directly but also indirectly on its targets. The involvement of STE12 in secondary metabolism likely includes an impact on iron homeostasis via siderophores, and a clear effect on the production of polyketide secondary metabolites in *T. reesei*. Hence, also considering the background of knowledge from other fungi, STE12 exerts important functions in primary and secondary metabolism, which are likely associated with balancing energy distribution between enzyme production, secondary metabolite production and development in response to given environmental conditions.

## Materials and methods

### Strains and cultivation conditions

*T. reesei* QM6a^73,74^ and QM6a∆*ku80* were used as parental strains in this study. To investigate gene regulation, enzymatic activity and biomass formation, liquid cultivation was performed under both continuous light and constant darkness conditions at 200 rpm and 28 °C for 96 h. Prior to inoculation, the strains were cultured on agar plates containing 3% (w/v) malt extract (MEX) in constant darkness for a period of 14 days to eliminate any potential effects of circadian rhythmicity. For the liquid culture, of 10^9^ conidia per liter were inoculated in Mandels-Andreotti minimal medium^75^ supplemented with 1% (w/v) microcrystalline cellulose (Alfa Aesar, Karlsruhe, Germany) as only carbon source. Additionally, 5 mM urea and 0.1% peptone were added to induce germination. Following the 96-h incubation, both mycelia and supernatants were collected and snap frozen in liquid nitrogen. In the case of cultures under constant darkness, only minimal red safety light was employed, specifically a darkroom lamp (Philips PF712E, red, 15W).

### Construction of the ste12 deletion strain

*Ste12* (TrA1391C/TR_36543) was deleted in QM6a∆*ku80* following the procedure described previously^76^ using yeast recombination and the hygromycin (hph) marker cassette. The protoplasting method was used for transformation and 50 µg/mL hygromycin B as selection reagent (Roth, Karlsruhe, Germany)^77^. Successful deletion was confirmed by the absence of the gene by PCR and primers 36543_qF and 36354_qR (Table 1). DNA integrity was confirmed by a parallel PCR with primers EF1-728F and TEF1_rev to avoid a false negative result. Copy number determination confirmed the single integration of the deletion cassette^78^.

**Table 1:** Oligonucleotides used in this study.

Primer name	Info	Sequence 5′–3′	Target gene	Notes
pdel_36543_5F	construction of deletion cassette	GTAACGCCAGGGTTTTCCCAGTCACGACGTGTACCTGTACCTTACCACG	*ste12*	This study
pdel_36543_5R	construction of deletion cassette construction of deletion cassette	ATCCACTTAACGTTACTGAAATCTCCAACGTGTGTGTGTGAGAGAGACC	*ste12*	This study
pdel_36543_3F	construction of deletion cassette	CTCCTTCAATATCATCTTCTGTCTCCGACTCCAGTGGGATAATACCTGC	*ste12*	This study
pdel_36543_3R	construction of deletion cassette	GCGGATAACAATTTCACACAGGAAACAGCTCTCCTATTACCTGTCTACG	*ste12*	This study
RT_36543_F	internal primer	CCACATCAGCGACGACAT	*ste12*	This study
RT_36543_R	internal primer	GAGTGAGACTTGTGAGGGTAAG	*ste12*	This study
EF1-728F	internal primer	CATCGAGAAGTTCGAGAAGG	*tef1*	^92^
TEF1 rev	internal primer	GCCATCCTTGGAGATACCAGC	*tef1*	^93^
RTcbh1F	qPCR primer	ACCGTTGTCACCCAGTTCG	*cbh1*	^94^
RTcbh1R	qPCR primer	ATCGTTGAGCTCGTTGCCAG	*cbh1*	^94^
RT_VEL_R1	qPCR primer	GCAGGAACACCAGTCAGGATG	*vel1*	^33^
RT_VEL_F1	qPCR primer	CGAGGAGGGCAAGGACATTAC	*vel1*	^33^
SAR RTF1	qPCR primer	TGGATCGTCAACTGGTTCTACGA	*sar*	^95^
SAR RTR1	qPCR primer	GCATGTGTAGCAACGTGGTCTTT	*sar*	^95^
RT_82208_F	qPCR primer	ACTGAAGCAGTATCGGGCAACT	*pks4*	^61^
RT_82208_R	qPCR primer	TCTTCGACGTAAAGAGCAGCCA	*pks4*	^61^
xyr1RTF	qPCR primer	CTTCCTCCTCCTGCTCATCG	*xyr1*	^96^
xyr1RTR	qPCR primer	TCGTGTGCCCTAACAATGGTC	*xyr1*	^96^
RT_CRE1 F	qPCR primer	GCAGCACAATACGACTCCG	*cre1*	This study
RT_CRE1 R	qPCR primer	CGGCTAATGATGTCGGTAAG	*cre1*	This study

### Isolation and manipulation of nucleic acids

The Qiagen RNeasy Plant mini kit was used for the isolation of RNA from mycelia from liquid culture. RT-qPCR was performed with three biological and three technical replicates as described previously^62,79^ using the GoTaq® qPCR Master Mix (Promega) as previously described with *sar1* as reference gene and other primers listed in Table 1. For mutant screening DNA was isolated following the rapid minipreparation protocol for fungal DNA as described previously^80^.

### Transcriptome analysis

Total RNA was provided in biological triplicates for every strain and condition. Sequencing and library-preparation using ribo-depletion to eliminate rRNA was performed at the Next Generation Sequencing Facility (Vienna Biocenter Core Facilities GmbH (VBCF), Austria). The sequencing was carried out on a NovaSeq 6000 platform using a paired-end (PE) configuration and 150 bp mode and yielded an average of 31 million reads per sample. Data analysis was performed as previously described^24^, briefly: Quality filtering (Q30) was done using bbduk version 38.18^81^, mapping to the most recent *T. reesei* QM6a reference genome^73^ was done using HISAT2 version 2.2.1^82^. Furthermore, samtools version 1.10^83^, QualiMap version 2.2.2^84^ and featureCounts version 2.0.1^85^ were used. Differential gene expression (DEG) analysis (DESeq2 version 1.3.1)^86^ was performed in R version 4.0.3 (https://www.R-project.org), with a threshold for significantly differentially regulated genes with log2fold change |> 1| and p-adj < 0.05. Gene annotations were performed employing existing annotations for *T. reesei*, *T. virens* and *T. atroviride*^87^ and *T. reesei*^88^. The DESeq2 variance stabilizing transformation (VST) function was applied for count normalization. Functional enrichment of a set of DEGs was performed using the Fisher’s exact test using R package topGO version 2.42.0 (https://bioconductor.org/packages/topGO) visualized with the R package rrvgo (p-value < 0.1, weighted algorithm 0.7 threshold)^89^. The specific script developed for and used in this analysis is available at: https://github.com/miriamschalamun/RNA_Tricho/tree/main”.

### Statistics

Statistical significance for RTqPCR, cellulase activity and biomass analysis was calculated in R using Student’s T-test (compare means, ggpubr version 0.4.0) ** = p-value < 0.01, * = p-value < 0.05.

### BIOLOG phenotype microarray analysis

Variations in growth based on diverse carbon sources were assessed using the BIOLOG FF Microplate assay (Biolog Inc., Hayward, CA), as described previously^90^. Inoculated microplates were incubated at 28°C in constant darkness, spanning a timeframe of up to 144 h. Measurements of absorbance at 750 nm, indicative of biomass accumulation, were taken at 24-h intervals, starting at 72-h. To evaluate the statistical significance of growth differences, a T-test was employed (with a threshold p-value of ≤ 0.05) using Excel 2016 (Microsoft, Redmond, USA).

### Secondary metabolite analysis

Secondary metabolites were extracted from strains grown on 3% malt extract medium in constant darkness for 14 days in triplicates as described previously^24,39^. Samples were prepared from each two agar plugs of 1.8 cm^2^ from 3 plates. Extraction was done in 15 mL tubes by adding 3 mL of 50% acetone in water (v/v) and ultrasonication for 15 min. Thereafter, 1 mL of chloroform was added. For phase separation, tubes were centrifuged at 4 °C at 1000 g for 1 min. The organic phase was transferred to glass vials and left for evaporation overnight. This step was repeated two times. The dry extracts were redissolved in 140 μL MeOH for HPLC analysis.

Analytical HPLC-UV-DAD measurements were done on Agilent 1100 series coupled with UV-diode array detection at 230 nm and a Hypersil BDS column (100 × 4 mm, 3 μm particle size). An aq. buffer containing 15 mM H_3_PO_4_ and 1.5 mM tetrabutylammonium hydroxide (A) and MeOH (B) were used as eluents. The following elution system was applied: From 55 to 95% B within 8 min, and 95% B was kept for 5.0 min, with a flow rate of 0.5 mL/min. The injection volume was 5.0 μL.

HR-ESI-TOF–MS spectra were obtained on a maXis UHR ESI-Qq-TOF mass spectrometer (Bruker Daltonics, Bremen, Germany). Samples were dissolved and further diluted in ACN/MeOH/H_2_O in the ratio of 99:99:2 (v/v/v) and directly infused into the ESI source with a syringe pump. The ESI ion source was operated as follows: capillary voltage: 4.0–4.5 kV, nebulizer: 0.4 bar (N_2_), dry gas flow: 4 L/min (N_2_), and dry temperature: 180 °C. Mass spectra were recorded in the range of *m/z* 50–1900 in the positive- and negative ion mode. The sum formulae of the detected ions were determined using Bruker Compass DataAnalysis 4.1 based on the mass accuracy (Δ*m/z* ≤ 5 ppm) and isotopic pattern matching (SmartFormula algorithm).

Sample A was dissolved in deuterated solvent (acetone-*d*_6_, 5 mg in 0.6 mL) and transferred into a 5 mm high precision NMR sample tube for NMR spectroscopic measurements. 1D and 2D NMR spectra were recorded on a Bruker AVIII 600 spectrometer (Bruker, Rheinstetten, Germany) at 600.13 MHz (^1^H) and 150.91 MHz (^13^C), respectively and processed with Topspin 4.1. Chemical shifts (δ) are reported in ppm; for ^1^H relative to residual acetone-*d*_5_ (δ_H_ = 2.05 ppm) as well as for ^13^C relative to acetone-*d*_6_, (δ_C_ = 29.8 and 206.3).

### Purification and identification of dehydroacetic acid

In the course of preparative isolation and purification of *T. reesei* secondary metabolites a lipophilic extract (696 mg) of mutant strains was suspended in approx. 5 mL of a mixture consisting of 30% *n*-heptane in ethyl acetate and adsorbed on 3 g silica gel 60 (0.2–0.5 mm grain size). After the solvent disappeared the dry silica gel powder was subjected to column chromatography over 24 g silica gel 60, 40–63 µm grain size, eluted with *n*-heptane: ethyl acetate mixtures in ratios of 95:5, 90:10, 85:15, 80:20, 75:25, 70:30 and 45:55 (100 mL each; fraction size 50 mL). The fractions eluted with 80:20 were pooled after HPLC analysis (38 mg) and subjected to size exclusion chromatography over Sephadex LH20 (GE Healthcare) eluted with acetone which afforded in total 6 mg of a mixture of the target compound and a steroid in higher quantities, determined by MS and NMR (sample A).

A Merck Silica gel 60 glass plate were used for preparative thin layer chromatography (TLC) to obtain sample B. This plate was developed in *n*-heptane/ ethyl acetate 70:30 (v/v).

### Supplementary Information

Supplementary Information 1.

Supplementary Information 2.

Supplementary Information 3.

## Data Availability

The datasets generated and analyzed during the current study are included in this article and its additional files and under GenBank accession number GSE222127 (https://www.ncbi.nlm.nih.gov/geo/query/acc.cgi?&acc=GSE222127).
