# Supplementary material for: The transcription factor STE12 influences growth on several carbon sources and production of dehydroacetic acid (DHAA) in Trichoderma reesei
Source: Sci Rep. 2024 Apr 26;14:9625. doi: 10.1038/s41598-024-59511-8 (PMC11053031; doi:10.1038/s41598-024-59511-8)
Supplement: Supplementary file 3 — Supplementary Information 3. [file 41598_2024_59511_MOESM3_ESM.pdf]

The transcription factor STE12 influences growth on several carbon sources and production of dehydroacetic acid (DHAA) in *Trichoderma reesei*

Miriam Schalamun[1], Wolfgang Hinterdobler[1,2], Johann Schinnerl[3], Lothar Brecker[4] and Monika Schmoll[1,5]*

[1] AIT Austrian Institute of Technology GmbH, Center for Health and Bioresources, Konrad Lorenz Strasse 24, 3430 Tulln, Austria
[2] MyPilz GmbH, Wienerbergstrasse 55/13-15, 1120, Vienna, Austria
[3] University of Vienna, Department of Botany and Biodiversity Research, Rennweg 14, 1030 Vienna, Austria
[4] University of Vienna, Department of Organic Chemistry, Währinger Strasse 38, 1090 Vienna, Austria
[5] University of Vienna, Department of Microbiology and Ecosystem Science, Division of Terrestrial Ecosystem Research, Djerassiplatz 1, 1030 Vienna, Austria

Supplementary material

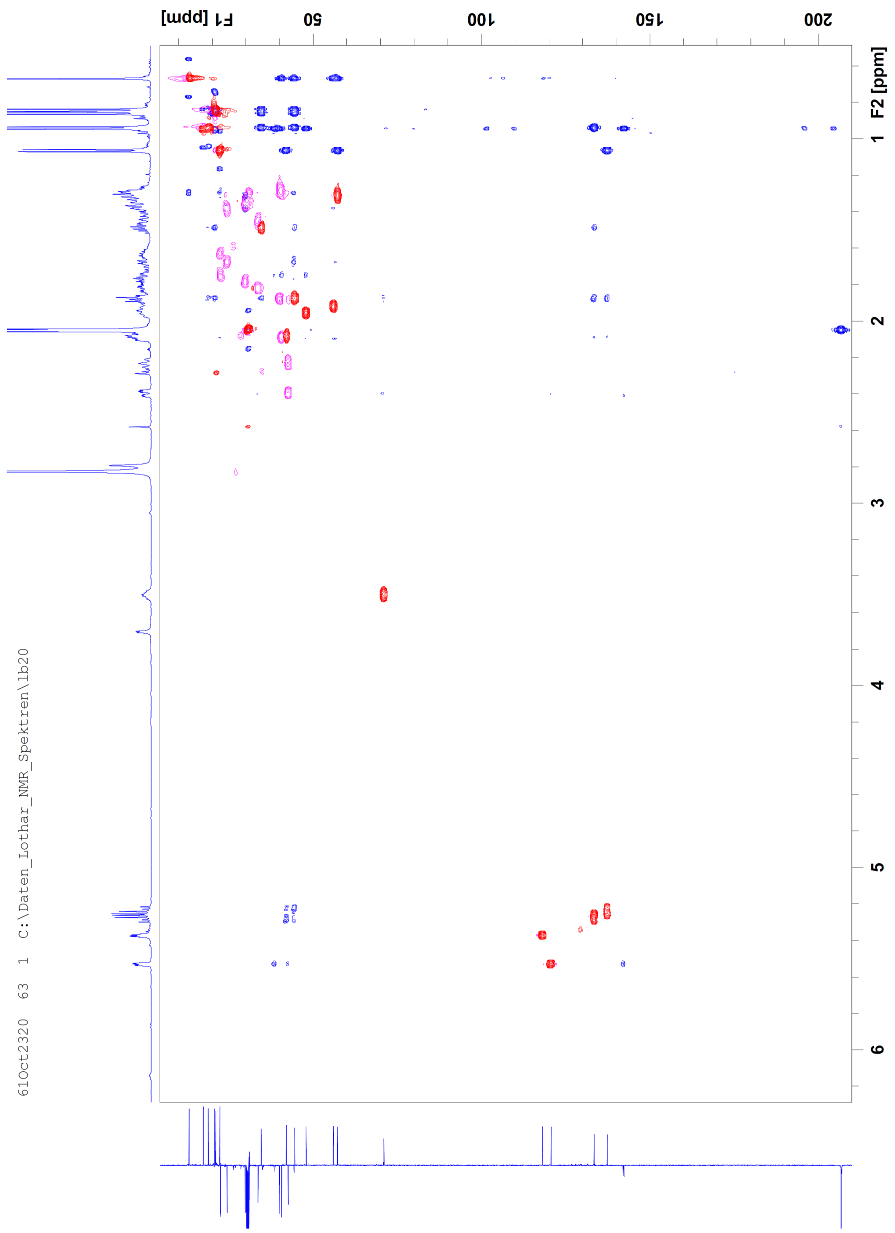

Figure S1: NMR spectroscopic measurements of sample A. Shown are HSQC [red (CH_3 / CH) and pink (CH_2)] as well as ^{1}H NMR and ^{13}C NMR spectra at the axes, respectively. Sample A was dissolved in acetone-d_6 and measured at 600.13 MHz (^{1}H) / 150.91 MHz (^{13}C). The threshold was chosen to mainly show the signals of the nuclei from ergosterol.

a)

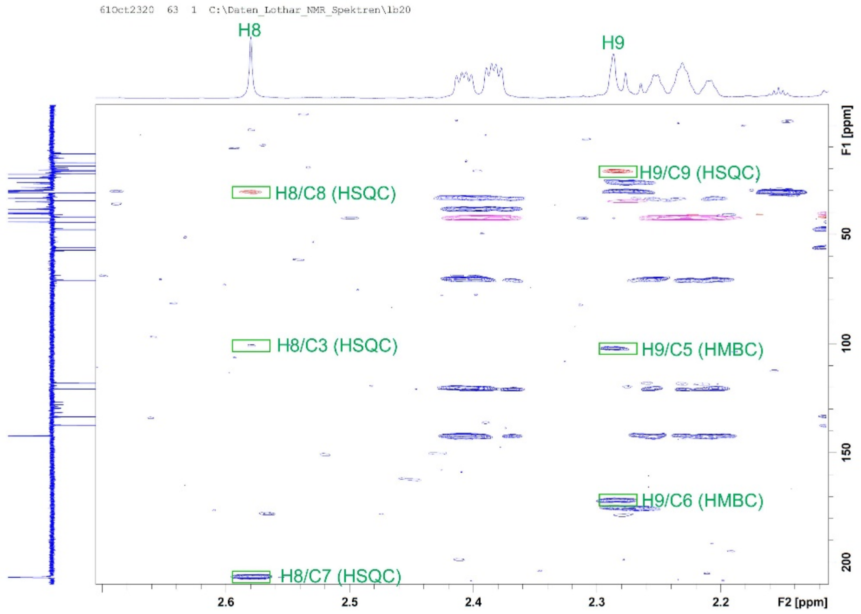

b)

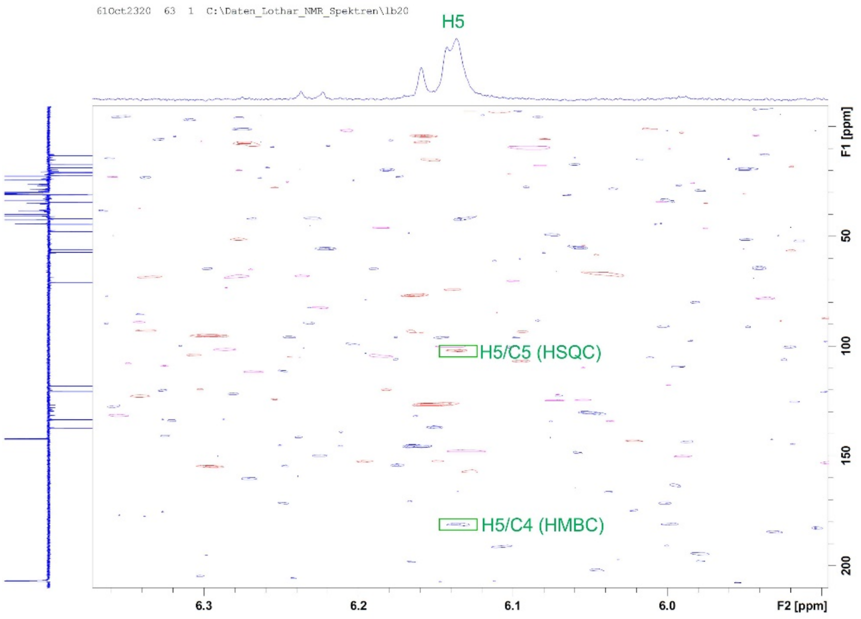

Figure S2: NMR spectroscopic measurements of sample A. Shown are HSQC [red (CH_3 / CH) and pink (CH_2)] as well as ^{1}H NMR and ^{13}C NMR spectra at the axes, respectively. Sample A was dissolved in acetone-d_6 and measured at 600.13 MHz (^{1}H) / 150.91 MHz (^{13}C). The cutouts and threshold were chosen to indicate the signals of the nuclei from dehydroacetic acid. The $^nJ_{H\text{-}C}$ couplings mentioned in the manuscript text for the structural analysis are indicated here by green boxes.

Generic Display Report

Analysis Info

Analysis Name	E:\Data\MS_MessService\74306000002.d	Acquisition Date	10/27/2020 3:46:28 PM
Method	tune_low_MS_Service_10_20.m	Operator	msc
Sample Name	DK-TR02	Instrument	maXis
Comment	Kokoric / Brecker<br>ACN/MeOH+1% H2O<br>Ergebnis +/- 5ppm		

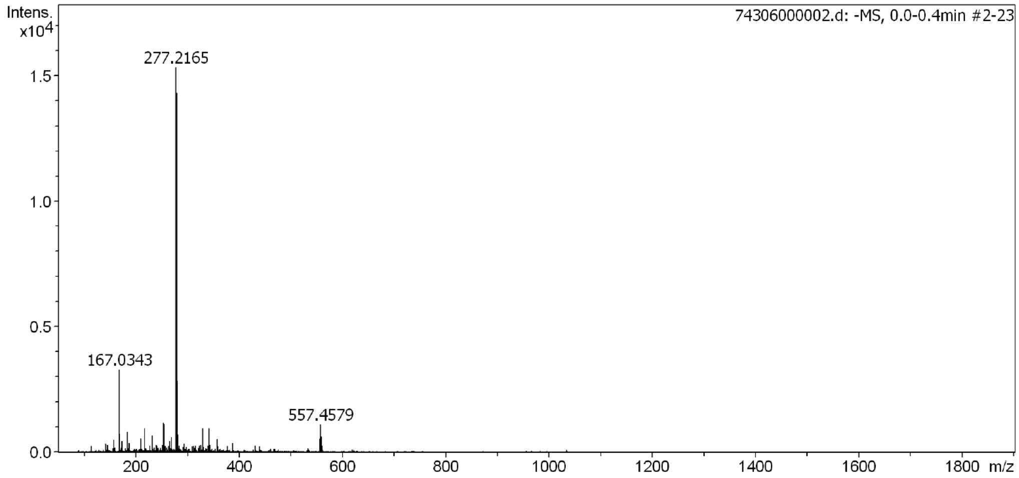

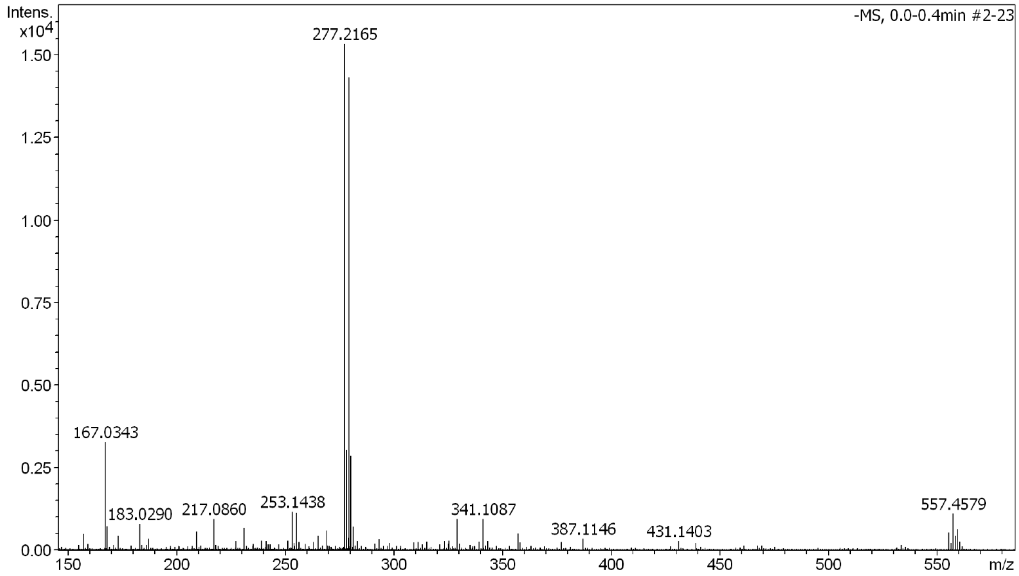

Bruker Compass DataAnalysis 4.1 printed: 10/27/2020 3:48:16 PM by: msc Page 1 of 1

Figure S3: HR ESI mass spectrum of sample A in negative ionization mode. The $[M-H]^-$ of *m/z* 167.0343 is assigned to the molecular formula $C_8H_8O_4$ of dehydroacetic acid.

Generic Display Report

Analysis Info

Analysis Name	D:\Data\MSC service\100392000002.d	Acquisition Date	10/18/2023 1:45:03 PM
Method	ESI_DI_standard_neg_2023.m	Operator	Demo User
Sample Name	DK-TR02A	Instrument	timsTOF fleX
Comment	Schinnerl / Brecker / Org.Chem.<br>Ergebnis: +/- 5 ppm<br>ACN / MeOH + 1% H2O		

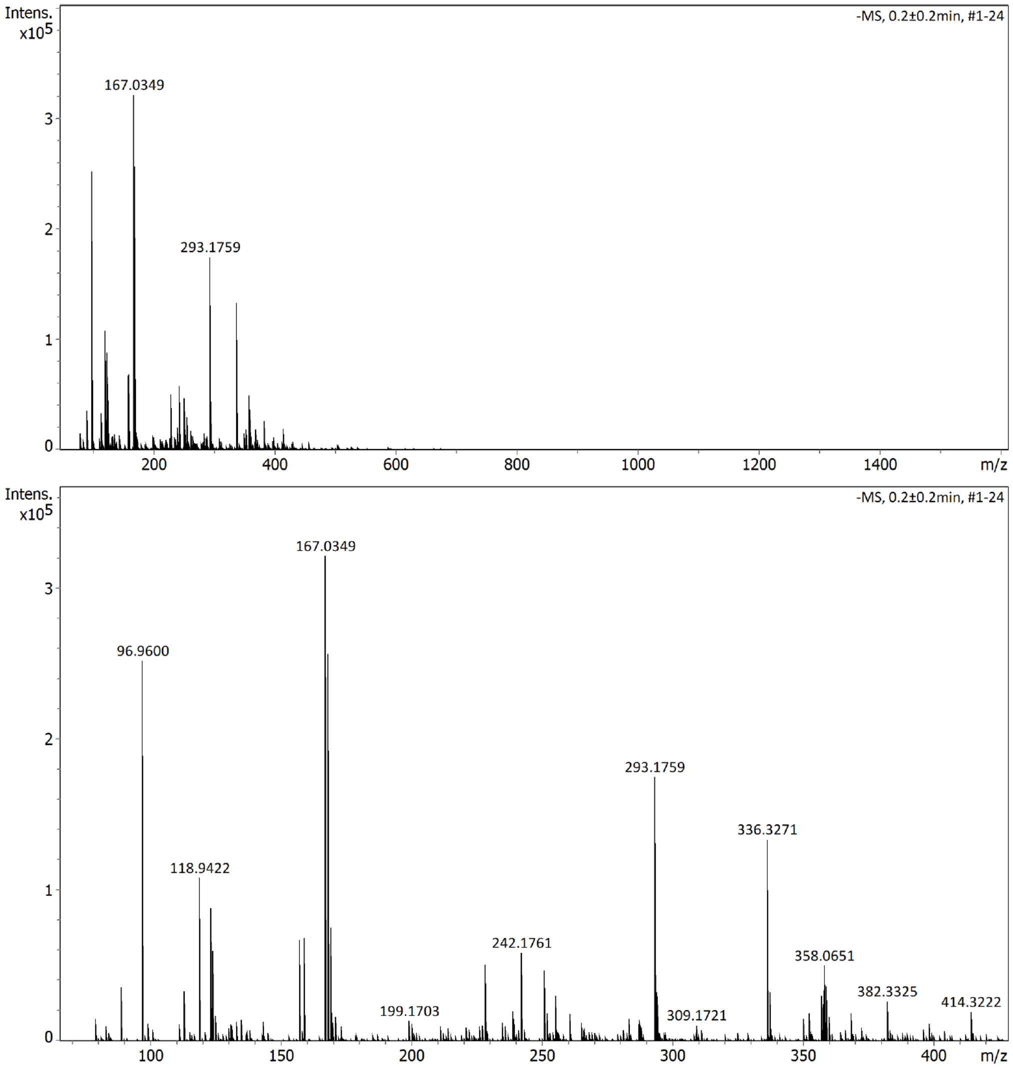

Bruker Compass DataAnalysis 5.3 printed: 10/18/2023 1:47:47 PM by: demo Page 1 of 1

Figure S4: HR ESI mass spectrum of sample B in negative ionization mode. The $[M-H]^-$ of *m/z* 167.0349 is assigned to the molecular formula $C_8H_8O_4$ of dehydroacetic acid. The isotopic pattern shows a weak entry of deuterium into the molecule, as it was previously dissolved in CD_3OD.

Generic Display Report

Analysis Info

Analysis Name	D:\Data\MSC service\100392000001.d	Acquisition Date	10/18/2023 12:24:55 PM
Method	ESI_DI_standard_2023.m	Operator	Demo User
Sample Name	DK-TR02A	Instrument	timsTOF fleX
Comment	Schinnerl / Brecker / Org.Chem.<br>Ergebnis: +/- 5 ppm<br>ACN / MeOH + 1% H2O		

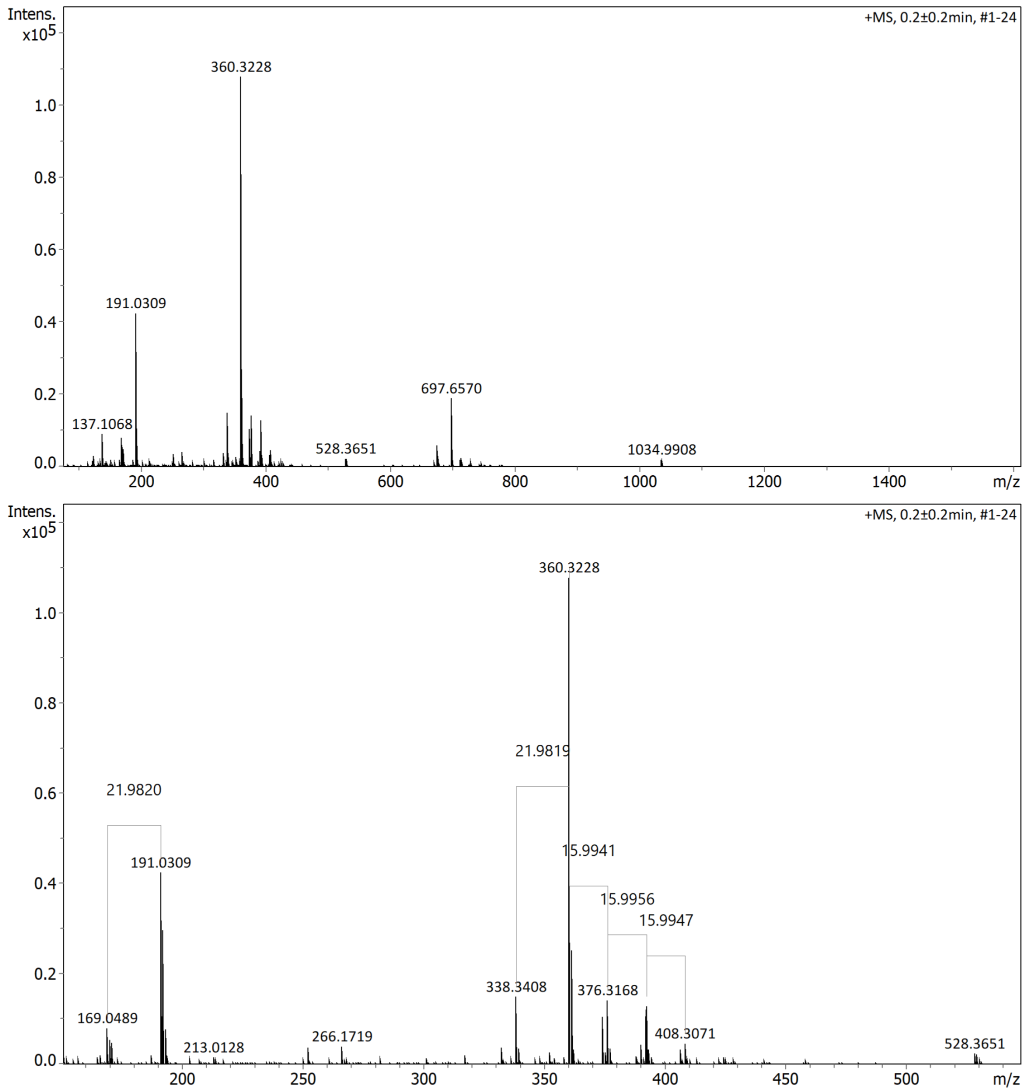

Bruker Compass DataAnalysis 5.3 printed: 10/18/2023 12:31:49 PM by: demo Page 1 of 1

Figure S5: HR ESI mass spectrum of sample B in negative ionization mode. The $[M+H]^+$ of *m/z* 169.0489 and the $[M+Na]^+$ of *m/z* 191.0309 are assigned to the molecular formula $C_8H_8O_4$ of dehydroacetic acid. The isotopic patterns show a weak entry of deuterium into the molecule, as it was previously dissolved in CD_3OD.

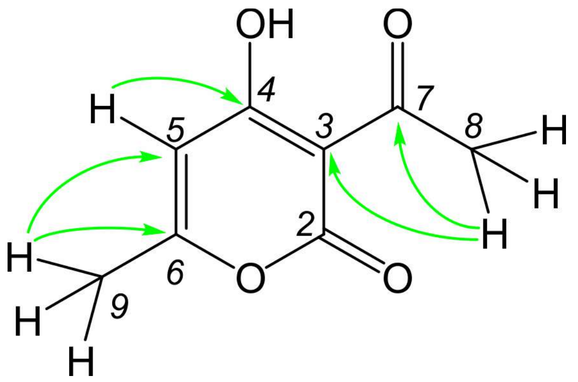

Figure S6: Molecular structure of dehydroacetic acid; only one possible isomer is shown. Numbering of position is in agreement with those in Table S1 and in the manuscript text. Indicative $^{2}J_{H\text{-}C}$ and $^{3}J_{H\text{-}C}$ coupling are visualized by green arrows.

Table S1: NMR chemical data of dehydroacetic acid from sample A, dissolved in acetone-d_6 and measured at 600.13 MHz (^{1}H) / 150.91 MHz (^{13}C).

	dehydroacetic acid	
position	δ_H (integral, multiplicity)	δ_C, multiplicity
2	-	162.4, s
3	-	100.9, s
4	-	180.4, s
5	6.14 (1H, s)	102.2, d
6	-	171.7, s
7	-	206.7, s
8	2.58 (1H, s)	30.7, q
9	2.28 (1H, s)	21.2, q

Numbering of positions is in agreement with those in Figure S6 and in the manuscript text.
